# What makes a doctor a scholar: a systematic review and content analysis of outcome frameworks

**DOI:** 10.1186/s12909-016-0627-z

**Published:** 2016-04-22

**Authors:** Stefanie C. Hautz, Wolf E. Hautz, Markus A. Feufel, Claudia D. Spies

**Affiliations:** Office of the Vice Dean for Teaching and Learning, Charité-Universitätsmedizin Berlin, Charitéplatz 1, 10117 Berlin, Germany; Department of Anesthesiology and Intensive Care Medicine, Charité Campus Virchow-Klinikum and Charité Campus Mitte, Charité-Universitätsmedizin Berlin, Berlin, Germany; Universitäres Notfallzentrum, Inselspital Bern, Bern, Switzerland

**Keywords:** Scholar, Scholarship, Outcome based education, Competency based education, Systematic review

## Abstract

**Background:**

Many national outcome frameworks (OF) call for a sound scholarship education and scholarly behaviour of physicians. Educators however are known to interpret the scholar role in markedly different ways and at least one major initiative to unify several national outcome frameworks failed to agree on a common definition of the scholar role. Both circumstances currently limit the development of educational and assessment strategies specific for the scholar role. Given increasing physician mobility together with the global perspective inherent in a doctor’s role as a scholar, we were interested in what different OFs define as the scholar role and attempted to identify communalities and differences between them.

**Methods:**

We conducted a systematic review for OF in medical education in PubMed and google. After in- and exclusion processes, we extracted all content listed under the scholar role (if present) and categorized it based on Boyer’s established model of scholarship. Next, we extracted all content related to scholarship from OFs not explicitly defining a scholar role and used it to validate the categories resulting from step one.

**Results:**

From 1816 search results, we identified 13 eligible OFs, seven of which explicitly specified a scholar role. The outcomes only partly map onto Boyer’s definition of scholarship: Discovery, Integration, Application, and Teaching. We adapted and validated a model extending this definition to contain Common Basics (partly overlapping with Integration and Teaching), Clinical Application (specifying Application), Research (Discovery and partly Integration), Teaching and Education (partly overlapping with Teaching) and Lifelong Learning (no equivalent in Boyer’s model). Whereas almost all OFs cover Common Basics, Clinical Application, and Lifelong Learning, fewer and less specific outcomes relate to Research or Teaching.

**Conclusions:**

The need to adapt existing models of scholarship may result from the changing demands directed at medical scholars. The considerable differences identified between OFs may explain why educators have difficulties defining the scholar role and why the role is rarely assessed. We may have missed OFs due to our in- and exclusion criteria but the results provide a solid basis on which to build a common understanding of what makes a doctor a scholar.

**Electronic supplementary material:**

The online version of this article (doi:10.1186/s12909-016-0627-z) contains supplementary material, which is available to authorized users.

## Background

Scholarship is a key activity for any academic health professional [1, 2]. In fact, many national regulations call for a sound scholarship education and scholarly behaviour of any physician, not just those at academic institutions [3, 4]. And although most educators would agree that “it is important to: (1) educate future physicians to be inquisitive; (2) help them build a strong scientific foundation for future medical practice; and (3) equip them with the knowledge, skills, and habits of mind to integrate new scientific discovery into their medical practice” [5], educators are known to interpret the scholar role in markedly different ways [6].

Furthermore, the Tuning Project, which aims to align outcome and competency definitions across frameworks from European countries, could neither agree on the definition nor on the specific competencies to be subsumed under the scholar role. Accordingly, the final document of the Tuning Project lists detailed outcomes for all roles but the scholar role [7]. Thus, defining the scholar role of a physician seems to be particularly challenging.

However, outcome-based education (OBE), the current gold standard in medical education, requires a clear definition of predetermined outcomes [8]. These outcomes inform all curricular decisions and strengthen the educational system’s accountability to the greater society. OBE has moved medical education “from the ‘how’ and ‘when’ to the ‘what’ and ‘whether’” [9]. It has previously been argued that outcomes (what the medical school expects of the graduate) and competencies (what the graduate is able to do) differ merely in the perspective taken [10]. In this article, we therefore use the two terms interchangeably and refer to both outcome-based and competency-based education as OBE.

As a prerequisite for OBE, many national associations and governmental bodies have developed outcome frameworks (OFs). These OFs are comprehensive collections of desired outcomes of medical education and define the competencies that physicians should possess when graduating from medical school and/or after residency [10]. Most OFs are subdivided into sections organized by the roles that graduates are expected to play in their professional lives. For example, the CanMEDS OF specifies seven roles: Medical Expert, Professional, Communicator, Collaborator, Manager, Health Advocate, and Scholar [11].

Consequently, a comparison of existing OFs definition of the scholar role should answer the question: What makes a doctor a scholar?

Independent of the discipline of medicine, Ernest Boyer, educator and former president of the Carnegie Foundation for the Advancement of Teaching, developed a generic model of scholarship [12]. He defines four domains of scholarship: Discovery, Integration, Application, and Teaching. By Discovery, he means original, active research that advances knowledge. Integration is the synthesis of information across disciplines (establishing connections across disciplines), topics (comprehensive understanding of research results), and time. Application refers to the application of disciplinary expertise to the individual or institution. The scholarship of Teaching in Boyer’s model covers knowledge of teaching and educating others (especially in critical thinking), which is implicitly assumed to result in learners recognizing the need for continues learning. Boyer’s model is widely cited in articles on scholarship in health professions education. Both relevant AMEE guides on scholarship in medical education are based on his definition [1, 13] and authorities ground their advice for the development of scholarly projects in Boyer’s model [2].

Advancing Boyers work, Glassick developed criteria to judge whether a given piece of work qualifies as scholarship [14]. These criteria include clear objectives, adequate methods, significant results, targeted communication, and reflective critique. However, whether and how Boyer’s model or Glassick’s criteria are reflected by current OFs definition of the scholar role is unknown. Furthermore, we are not aware of a consistent definition of the scholar role, or of scholarship, within and for medical education.

Given the importance of clearly defined roles as guideposts within and for OBE and the evident difficulties in defining the scholar role, we compared existing OFs to identify similarities and differences in their definitions of the scholar role. Specifically, we asked what constitutes the scholar role of a medical doctor as defined by the OFs. In doing so, we aim to advance the comparability of the doctors’ role as a scholar between frameworks, the development of suitable assessment instruments, and the mobility of graduates between different OFs ‘jurisdiction’.

Because Boyer’s model of scholarship is frequently cited in publications on scholarship in medical education, we hypothesized that all content of the scholar role as defined by the OFs could be mapped to this model. We thus used its four categories of Discovery, Integration, Application, and Teaching to guide our qualitative content analysis of current OFs.

## Methods

To extract and compare definitions of the doctor as a scholar from different OFs, we followed a three-step approach consisting of search for OFs, in- and exclusion, and data extraction, categorization, and validation. Search for and in- and exclusion of OFs have been previously described elsewhere in detail [15] and are summarized here briefly.

### Search for OFs

**Fig. 1:**
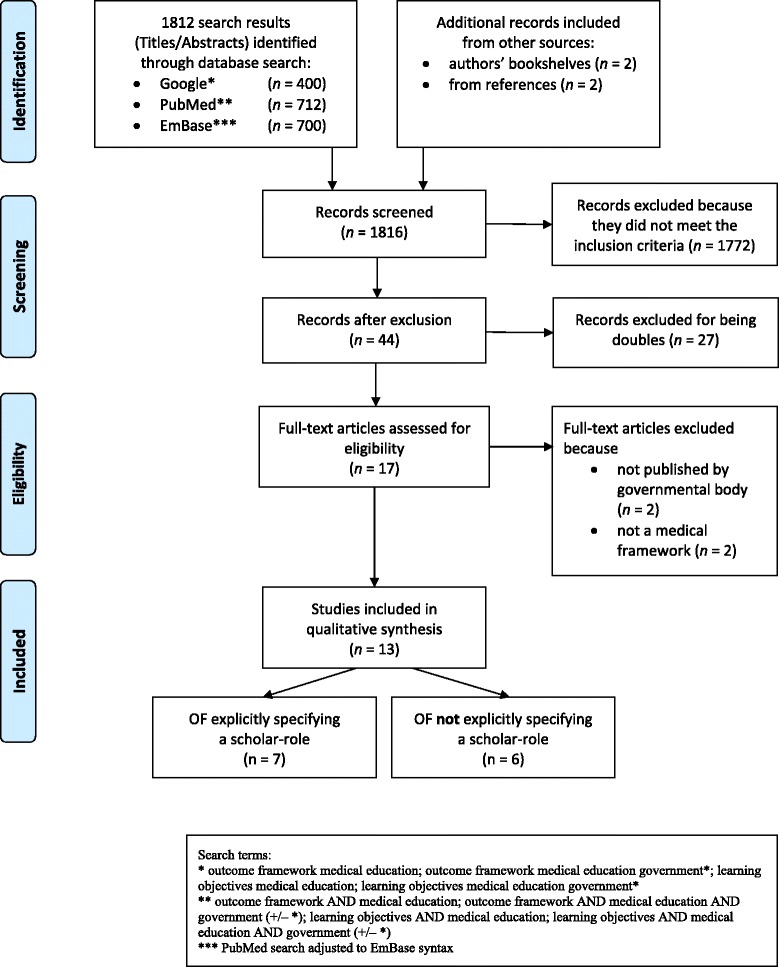
Selection of OFs

To develop keywords and assess the quality of our search terms, we first identified six well-known OFs in medical education [3–5, 11, 16–18] as a control sample. In June 2013, a librarian with expertise in searching biomedical databases and one of the authors (SH) searched electronic databases (PubMed and EmBase) and the Internet (http://www.google.com) for OFs published since January 1980. We refined the keywords used until all six OFs in the control sample were retrieved by our searches. The final search terms included medical education, outcome framework, learning objective, government*, curriculum, and combinations thereof (* used as wildcard to include government, governmental etc., see Fig. 1) [19]. The complete search strategy is available upon request.

Despite its less transparent search algorithms, an Internet search was included because OFs are not necessarily published in journals referenced in PubMed or EmBase, and any such OFs would thus be only accessible via Internet search engines. For the same reason, we also searched all references cited in the included OFs. For each database search, we retrieved all hits; for the Google search, we retrieved the first 100 hits, as the relevance of results decreased sharply thereafter.

### In- and exclusion

Two of us (SH, WH) scanned all results independently (noting relevant OFs using Google Bookmarks) and saved the results in OneNote. We created bookmarks and folders to archive web links and used ReferenceManager12 to create a bibliographic database. The search strategy and the total number of OFs retrieved are shown in Fig. 1. We included all OFs based on a national or international process and endorsed or published by a national or international society or governmental body. We excluded OFs from disciplines other than medicine or from medical specialities, as well as inter-professional frameworks and student-developed frameworks. Based on these criteria, inter-rater agreement (SH, WH) for the identification of relevant OFs was 97.68 %. Conflicts were resolved by discussion to reach a consensus. In a second round, we eliminated doubles from the remaining set of frameworks (100 % agreement). We did not in- or exclude OFs based on their content, but solely on the formal criteria stated above.

### Data extraction, categorization, and validation

We identified two general types of eligible OFs: those that explicitly specified a scholar role (*n* = 7) and those that did not (*n* = 6).

**Fig. 2:**
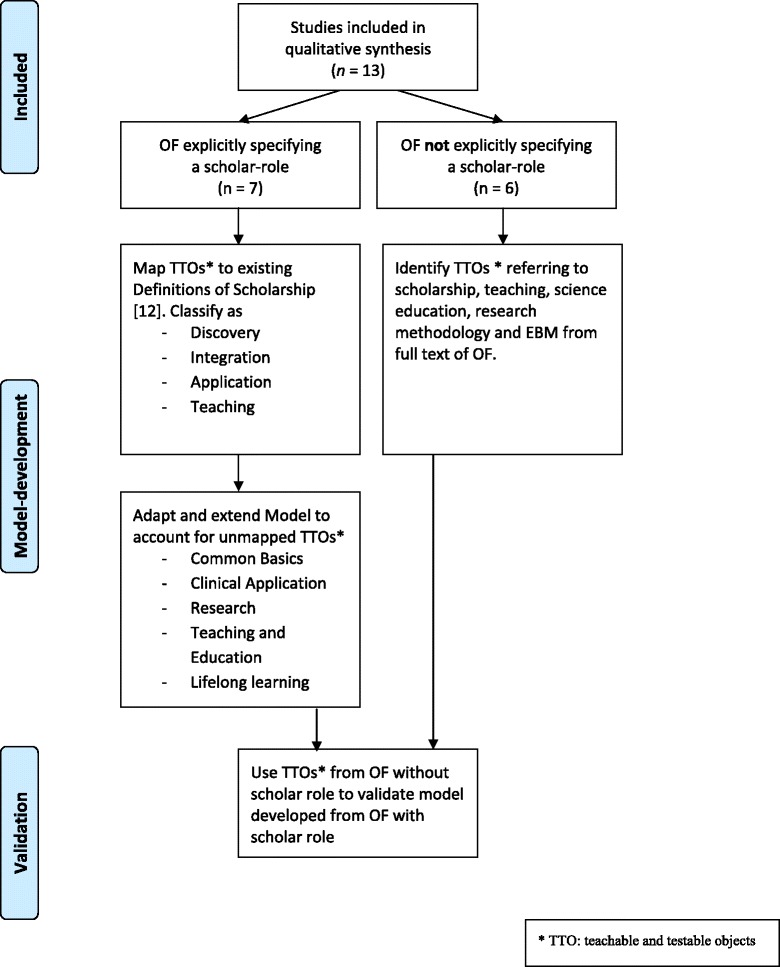
Analysis of included OFs

In a first step, we used the seven OFs that explicitly specified a scholar role to generate a common model of the doctor as a scholar. We intentionally drew on the existing definitions to reduce subjectivity in developing a model for the scholar role: We included only what others (the authors of the respective OFs) summarized under the term scholar. In a second step, we used data from the six OFs that did not explicitly specify a scholar role to validate the model resulting from the first step (see Fig. 2 and below: Validation of the model).

All included OFs contain objectives (e.g., competencies or outcomes) but use various terms to describe these educational targets, such as learning objectives, outcomes, competencies, general objectives, and attributes. Some OFs even combine two or three of these terms, usually without a clear definition or distinction. To minimize confusion, we refer to all these educational targets herein as teachable or testable objects (TTOs).

#### Categorization

We first extracted the definitions of the scholar role from the seven OFs that specified such a role. We then sought to map the contents of these definitions onto Boyer’s model [12] with its four components of scholarship: Discovery, Integration, Application, and Teaching. In doing so, we intentionally employed a directed approach to qualitative content analysis, detailed below (for a review, see e.g., [20]). If the raw material included more than one item (e.g., outcome or competency) per sentence, we separated the items into single TTOs to facilitate mapping to categories.

Not every aspect of the raw material could be allocated to one of Boyer’s four components. We therefore adapted and extended Boyer’s model by inductively categorizing the raw material. Two of us (SH, WH) used an iterative process to assemble, disassemble, and re-assemble all of the raw material with the aim of summarizing all extracted TTOs within one adapted model.

The guiding principles for the categorization of outcomes were (1) to categorize concordant outcomes together, (2) to establish as few categories as possible but as many as necessary to be able to assign all outcomes to exactly one category, and (3) to create face-valid categories with immediate relevance to the doctor as a scholar (medical scholarship), starting from Boyer’s [12] universal definition of scholarship (Fig. 2, left side). In this way, we developed a model whose categories and subcategories covered all outcomes extracted. Disagreements in assigning outcomes to a category were resolved by discussion.

#### Validation of the adaption of Boyers model

To validate the resulting model, we extracted all TTOs referring to scholarship, teaching, science education, research methodology, and evidence-based practice from all domains and roles of the six OFs that did not specify a distinct scholar role. We then sought to match these contents to the categories generated in the previous step (Fig. 2, right side). Three of us (SH, WH, MF) evaluated the mapping of these contents and their fit to the model. Disagreements were again resolved by discussion.

Throughout the analytic process, the source of all TTOs was logged so that we were able to compare the identified categories against the source OF, the number of OFs contributing to each category, the number of outcomes per category, and the roles (scholar role versus no scholar role) contributing to each category (see Additional file 1: Online Appendix for a complete list of all TTOs extracted and their assigned category in the final model). The results reported her are based on the “Standards for reporting qualitative research” [21].

## Results

### Characteristics of the OFs included

In total, our search yielded 1,816 hits. Of these, 44 remained for full analysis after initial screening of title and abstract. After we had eliminated 27 doubles and 4 results that proved not to be eligible when we examined the full text, 13 unique frameworks remained for analysis, of which seven explicitly specified a scholar role (see Tables 1 and 2).

**Table 1:** Included outcome frameworks explicitly specifying a scholar role

Name	Definition of the scholar	Target audience	Source
Accreditation Standard for Primary Medical Education Providers and their Program of Study and Graduate Outcome Statements, Australia, New Zealand.	“The medical education provider is active in research and scholarship, which informs learning and teaching in the medical program”.“3.2.1 Science and Scholarship: The medical graduate as scientist and scholar. The curriculum includes the scientific foundations of medicine to equip graduates for evidence-based practice and the scholarly development of medical knowledge”.	UG	[22]
Blueprint 2001: Training of doctors in the Netherlands, Netherlands.	“The doctor is scientifically educated and acts accordingly. This distinguishes him from not academically trained health professionals. He is acquainted with the basic principles of scientific research, not only in the form of scientific knowledge, but also in the form of practical experience through active participation in a scientific research project. He is able to approach scientific data critically and form independent opinions. He has reasonable insight regarding the extent of scientific underpinning of medical practice or of the absence of a scientific basis. He can verify the scientific underpinning of medical actions and communicate information to others”.	UG	[16]
CanMEDS Framework, Canada.	“As Scholars, physicians demonstrate a lifelong commitment to reflective learning, as well as the creation, dissemination, application and translation of medical knowledge”.	UG/PG	[3]
The Tuning Project, Learning Outcomes/Competences for Undergraduate Medical Education in Europe, EU.	“[We] leave it open to individual countries, schools or students to decide how to prioritize practical research experience, in keeping with their profile, educational philosophy or career intentions”.	UG	[7]
Swiss Catalogue of Learning Objectives for Undergraduate Medical Training, Switzerland.	“At the end of undergraduate education and the beginning of postgraduate training physicians engage in a lifelong pursuit of mastery of their domain of professional expertise. They recognize the need to be continually learning”.	UG	[18]
The Scottish Doctors: Learning Outcomes for the Medical Undergraduate in Scotland: A Foundation for Competent and Reflective Practitioners, Scotland.	“The competent graduate recognizes, explains and manages health problems using the principles of current scientific knowledge and understanding that underpin all of medicine”.	UG	[17]
Tomorrow’s Doctors. Outcomes and Standards for Undergraduate Medical Education, UK.	“§8: The graduate will be able to apply to medical practice biomedical scientific principles, method and knowledge relating to: anatomy, biochemistry, cell biology, genetics, immunology, microbiology, molecular biology, nutrition, pathology, pharmacology and physiology.	UG	[4]
	§9: Apply psychological principles, method and knowledge to medical practice.		
	§10: Apply social science principles, method and knowledge to medical practice.		
	§11: Apply to medical practice the principles, method and knowledge of population health and the improvement of health and healthcare.		
	§12: Apply scientific method and approaches to medical research”.		

*UG* undergraduate, *PG* post graduate

**Table 2:** Included outcome frameworks not explicitly specifying a scholar role

Name	Target audience	Source
Australian Curriculum Framework for Junior Doctors, Australia.	PG	[23]
Good Medical Practice, UK.	PG	[24]
A Guide to Good Medical Practice, USA.	PG	[25]
Developing a Framework of Competencies for Medical Graduate Outcomes, Australia, New Zealand.	UG	[26]
Report 1 - Learning Objectives for Medical Student Education, Guidelines for Medical Schools, USA.	UG	[27]
Visions 2015, India.	UG/PG	[28]

*UG* undergraduate, *PG* post graduate

All of the OFs included in our analysis contain content related to scholarship. We identified a total of 268 TTOs from the 13 OFs included in the analysis (see Additional file 1: Online Appendix).

### A model of the doctor as scholar

Because we hypothesized that the content of all scholar roles could be mapped onto Boyer’s [12] universal model of scholarship, we aimed to allocate each TTO to the category of either Discovery, Integration, Application, or Teaching. This approach left us with some unmatched content: Medical scholarship seems to have some peculiar requirements that necessitate the adaption and extension of Boyer’s universal model.

We therefore iteratively developed a five-component model from the seven OFs that explicitly specified a scholar role, adding one more component and redefining Boyer’s four categories to describe the TTOs identified more precisely: Common Basics, Clinical Application, Research, Teaching and Education, and Lifelong Learning.

In this resulting model, each of the five components of the scholar role is subdivided into specifications which, in turn, contain the respective TTOs. The complete model, including all 268 TTOs together with their source framework, is available as an Additional file 1: Online Appendix. Table 3 presents all components and their specifications together with exemplary TTOs.

**Table 3:**
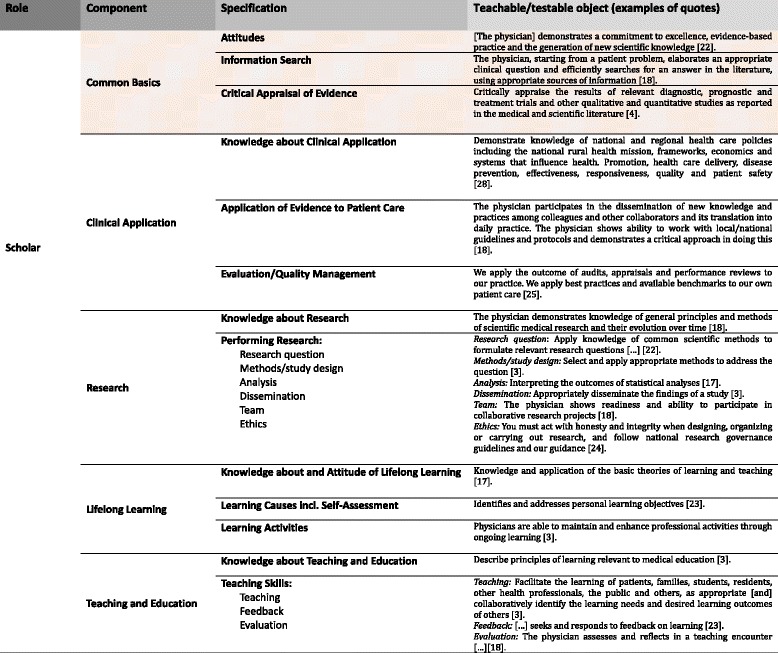
The medical scholar (shaded area: overarching component, see Results)

Common Basics comprises TTOs that are fundamental to all four other components. This component overlaps partly with Boyer’s categories of Integration and Teaching (e.g., comprehensive understanding). Each of its specifications—Attitudes, Information search, and Critical appraisal of evidence—was found in each of the other four components and recurred consistently.

The other four components are Clinical Application (specifying Boyer’s Application category), which describes the application of evidence to patient care, Research (Discovery and part of Integration in Boyer’s model), which encompasses knowledge about and competencies necessary for conducting research, Teaching and Education, and Lifelong Learning. Boyer’s category Teaching, which covers teaching, lifelong learning, and to some extent critical thinking, was split into two categories in our model, because each OF contained numerous references to teaching and lifelong learning. It seems that lifelong learning has gained importance within the medical profession over time.

The TTOs of all six OFs that do not explicitly specify a scholar role can also be categorized to these five components, thus validating the model. Only minor refinements of 2 subcategories were necessary to accommodate the scholar-related content of the six OFs without a specific scholar role to the model based on the seven OFs with such a role.

### Further findings

During the categorization process, it became evident that the level of detail of TTOs varied considerably across the components. Whereas outcomes mapped to Clinical Application were detailed and related to practice (e.g., “Applies the concept of specificity, sensitivity, pre- and post-test probability to the interpretation of common diagnostic procedures” [26]), most outcomes mapped to the components Research or Teaching and Education were less detailed and measurable (e.g., “Recognizing the important role of all doctors as mentors and teachers” [17]). Similarly, although every OF contains TTOs related to Lifelong Learning, outcomes in this category were vague compared with those in the Clinical Application category (e.g., “They recognize the need to be continually learning” [18]).

The components of the scholar role identified not only differed in the level of detail of the constituting TTOs. They also differed in the number of TTOs they contained, as well as in the number of OFs contributing to each component (see Table 4). With one exception, all OFs contained content that could be assigned to the components Clinical Application, Lifelong Learning, and Common Basics in our model. However, fewer OFs contained content relating to the components Research or Teaching and Education. Accordingly, the number of TTOs allocated to the component Teaching and Education was lower than that allocated, for instance, to the component Clinical Application (see Table 4).

**Table 4:** TTOs per component

Component of the scholar role	% of all TTOs^a^	Number of OFs (of the 13 included in the analysis) contributing TTOs to this component
Common Basics	22 %	12
Clinical Application	27 %	13
Research	19 %	11
Lifelong Learning	20 %	13
Teaching and Education	12 %	10

^a^ *TTOs* teachable or testable objects

## Discussion

Generally speaking, national OFs cannot simply be transferred to other countries: They are mostly context dependent and differ in important formal and structural characteristics [15]. Careful consideration is needed in adapting them to other contexts [29]. Yet unlike some roles that are more culturally sensitive (e.g., Communicator; see [30]), the role of the doctor as a scholar is grounded in a set of globally accepted research methodologies, study designs, teaching formats, publication standards, and means of evaluating scholarship. In particular, all content relating to research competence should be comparable across OFs, as scholarship is a key factor in the career advancement of (academic) health professionals worldwide [1, 2] and “research has traditionally crossed boundaries” [31]. Although there is disagreement among educators on whether the unity of research and teaching is a necessary condition for good education [32, 33], there is a trend toward integrating science- and research-education in undergraduate medical education [10]. This further highlights the need for a common definition of the scholar role.

We hypothesized all content extracted from the OFs included in the analysis to be categorizable to Boyer’s well-established generic model of scholarship—that is, to the categories of Discovery, Integration, Application, or Teaching [12]. Having extracted outcome definitions and TTOs from the seven OFs that explicitly specified a scholar role, we however found it necessary to adapt Boyer’s model to a model of medical scholarship in order to accommodate all extracted content. We were able to develop and validate the resulting model with the six OFs that did not explicitly specify a scholar role.

The need to extend and adapt Boyer’s model may result from the changing demands directed at medical scholarship. Whereas Boyer’s components of Discovery and Integration (summarized in our component Research) and Application (Clinical Application in our model) needed only minor changes to accommodate the outcomes specified in the included OFs, Teaching (Teaching/Education and Lifelong Learning in our model) seems to have gained importance, at least in the OFs included in our analysis. In particular, Lifelong Learning is given much higher priority nowadays. Interestingly, although all OFs included in this review contain content concerned with Lifelong Learning and explicitly mention Lifelong Learning in their definition of the scholar role (e.g., [3, 5]), we are not aware of any other published definition of scholarship that explicitly includes Lifelong Learning with such high priority. The results of our review suggest that, at least in the OFs included in this analysis, Lifelong Learning skills are integral to the physician’s role as a scholar.

As a second result, the content summarized under Common Basics has gained importance in comparison to Boyer’s model. For instance, information search skills and the use of new technologies now define students’ everyday work [34, 35].

Although it was possible to match all scholar-related content from all included 13 OFs to one of our five components, we found considerable differences between OFs in terms of the content covered and the level of detail and specificity. These differences may explain why medical educators have difficulties defining the scholar role [6] and why members of the Tuning Project could not agree on a common definition of the scholar role [7]. The differences within OFs (level of operationalization of TTOs) also make it difficult to compare or assess these scholarship-related outcome definitions. This may further explain why the role of scholar is much less frequently (or never) assessed in competency-based assessments [36], although frameworks for assessment based on outcome frameworks do exist [37].

Therefore, a content-based comparison and common definition of the scholar role such as the one developed within this paper could help to reduce or overcome these problems. The Additional file 1: Online Appendix summarizes all content related to the doctor as a scholar extracted from the OFs included in this study. It summarizes what the developers of 13 OFs define as the doctors role as scholar within an adapted existing model [12]. With this, we aim to foster the development of transferrable educational strategies for as well as assessment formats of the doctor’s role as scholar and its minimal consensus, found within this study. Such strategies and formats are especially necessary because medicine, as a globalizing profession, requires internationally agreed standards to guarantee quality medical education and, ultimately, quality care [31, 38]. The increasing mobility of medical personnel further intensifies the need for standardization and quality assurance mechanisms [38] for mutual degree recognition.

### Limitations

Although we supplemented the search for OFs in literature databases with an Internet search (because not all OFs are published in journals that are referenced in databases), we may have missed other eligible frameworks. Moreover, although we did not limit our search to specific countries, we only included frameworks published in English or German.

We did not include the ACGME outcome framework because it was unavailable at the time of our literature search and is in the process of getting reframed in the “milestones project”.

Furthermore, we only analysed the full text of the six OFs that did not specify a scholar role. We may have missed relevant content in the seven OFs that did explicitly specify a scholar role if that content was contained in some other part of the framework. We created this limitation on purpose, however, as we were interested in what others (the authors of the included OFs) classify as scholarship, more than our own opinion.

Finally, we did not differentiate between undergraduate and postgraduate learning outcomes, as many of the OFs reference each other as sources, irrespective of differences in the target population [15].

## Conclusion

Drawing on our comparison of 13 national OFs, we used existing definitions to develop a model of the scholar role in medical education. This model mirrors the contemporary requirements of medical scholarship as reflected in current OFs. The identified outcome components provide an evidence-based starting point to develop a common understanding of “the doctor as a scholar” and may help to increase the quality of curriculum development, implementation, and evaluation.

### Availability of data and materials

Complete raw data is available as supplemental online material. All further inquiries should be addressed to the corresponding author.

### Ethical approval

Not applicable.

## Additional file

Additional file 1: Online Appendix. (DOC 415 kb)
