# Supplementary material for: What makes a doctor a scholar: a systematic review and content analysis of outcome frameworks
Source: BMC Med Educ. 2016 Apr 22;16:119. doi: 10.1186/s12909-016-0627-z (PMC4841044; doi:10.1186/s12909-016-0627-z)
Supplement: Additional file 1: — Online Appendix. (DOC 415 kb) [file 12909_2016_627_MOESM1_ESM.doc]

| **Online Appendix** | | | | | |
| --- | --- | --- | --- | --- | --- |
|  | | | | | |
| **Common Basics for Scholars** (n = 12)  quotes: 59 | | | | | |
| **Attitude** (n = 7)  **Out of frameworks with a scholar-role:**  The doctor demonstrates a critical attitudes towards the scientific knowledge on which medical actions are based. (Metz, Verbeek-Weel & Huisjes, 2001)  …is scientifically educated: The doctor is scientifically educated and acts accordingly. This distinguishes him from not academically trained health professionals. (Metz, Verbeek-Weel & Huisjes, 2001)  He is able to approach scientific data critically and form independent opinions. (Metz, Verbeek-Weel & Huisjes, 2001)  Describes the principles of critical appraisal. (Frank, Jabbour & CanMEDS Working Group, 2005)  The physician demonstrates understanding that decisions in medical practice often are complex and based on uncertainty and probability data. (Working Group of the Swiss Medical Schools, 2008)  The physician seeks out opportunities to discuss the evidence base of clinical decision making and clinical care with colleagues and other health professionals. (Working Group of the Swiss Medical Schools, 2008)  Demonstrates a commitment to excellence, evidence based practice and the generation of new scientific knowledge. (AMC, 2013)  Respect for every human being, including respect of sexual boundaries. (Carmichael & Hourn, 2011)  **Out of all other domains:**  Honesty in representations :  - do our best to ensure that any documents we sign and testimony we provide are accurate, clear and verified;  - do not deliberately omit relevant information; (National Alliance for Physician Competence, 2009)  …graduates must appreciate the role of informatics in the day-to-day care of patients and advancement of medical science in general. (Scottish Deans' Medical Education Group, 2008  Adopting an inquisitive and questioning attitude where appropriate and applying rational processes. (Scottish Deans' Medical Education Group, 2008 | | **Information Search** (n = 10)  **Out of frameworks with a scholar-role:**  Reviewing the professional literature. (Metz, Verbeek-Weel & Huisjes, 2001)  Active search for relevant literature (e.g. library information and communicate technology systems). (Metz, Verbeek-Weel & Huisjes, 2001)  Making selections from the relevant professional literature and keeping abreast of the literature. (Metz, Verbeek-Weel & Huisjes, 2001)  Find information that is of professional interest in the literature and other sources and evaluates this information and transmit is to others. (Metz, Verbeek-Weel & Huisjes, 2001)  Be able to search (including through electronic means), and critically evaluate the medical literature and apply the information in the care of the patient. (Medical Council of India, 2011)  Conduct a systematic search for evidence. (Frank, Jabbour & CanMEDS Working Group, 2005)  Access and interpret the relevant evidence. (Frank, Jabbour & CanMEDS Working Group, 2005)  The physician, starting from a patient problem, elaborates an appropriate clinical question and efficiently searches for an answer in the literature using appropriate sources of information. (Working Group of the Swiss Medical Schools, 2008)  **Out of all other domains:**  Access information sources and use the information in relation to patient care, health promotion, giving advice and information to patients, and research and education. (General Medial Council, 2009)  Make effective use of computers and other information systems, including storing and retrieving information. (General Medial Council, 2009)  Efficiently access relevant information for patient management, research and ongoing professional development using information technology. (Carmichael & Hourn, 2011)  Identifies information, community resources and other resources which could assist with the management plan. (Carmichael & Hourn, 2011)  Collecting, storing and using information has always been an integral part of the practice of medicine.  It is now more complex and technology-based, thereby creating an increasing need for medical graduates to be competent in information handling skills ranging from simple record-keeping to accessing and using computer-based data. As well as having the technical skills to undertake such tasks… (Scottish Deans' Medical Education Group, 2008  Access data sources: Using library and on-line information sources, including internet and intranet systems accurately, systematically and in sufficient depth. (Scottish Deans' Medical Education Group, 2008  Use information and information technology effectively in a medical context´:  - use computers  - access information sources  - store and retrieve information (Cumming & Ross, 2008)  - use information and information technology effectively in a medical context (Cumming & Ross, 2008)  - define and carry out an appropriate literature search (Cumming & Ross, 2008)  Elaborating a clinical question and searching for its solution in the literature (Working Group of the Swiss Medical Schools, 2008)  The physician searches, collects, organizes and interprets health and biomedical information from different databases and sources. (Working Group of the Swiss Medical Schools, 2008)  …effectively utilizes information technology for patient care, self-learning and other activities (Working Group of the Swiss Medical Schools, 2008)  - We: seek timely answers to questions that arise at the time of care using appropriate information sources and databases; (National Alliance for Physician Competence, 2009)  - The ability to retrieve (from electronic databases and other resources), manage, and utilize biomedical information… (MSOP Report Writing Group, 1998)  How to find […] evidence. (Scottish Deans' Medical Education Group, 2008 | | **Critical Appraisal of Evidence** (n = 10)  **Out of frameworks with a scholar-role:**  Critically appraise the results of relevant diagnostic, prognostic and treatment trials and other qualitative and quantitative studies as reported in the medical and scientific literature. (General Medial Council, 2009)  He can verify the scientific underpinning of medical actions.  He has reasonable insight regarding the extent of scientific underpinning of medical practice or of the absence of a scientific basis. (Metz, Verbeek-Weel & Huisjes, 2001)  Critically appraise retrieved evidence in order to address a clinical question. (Frank, Jabbour & CanMEDS Working Group, 2005)  Critically evaluate medical information and its sources, and apply this appropriately to practice decisions. (Frank, Jabbour & CanMEDS Working Group, 2005)  Access, critically appraise, interpret and apply evidence from the medical and scientific literature. (AMC, 2013)  The physician demonstrates knowledge of principles of critical appraisal… (Working Group of the Swiss Medical Schools, 2008)  …apply basic principles of critical appraisal to sources of medical information. This involves developing scientific curiosity and use of scientific evidence in clinical decision making. (Working Group of the Swiss Medical Schools, 2008)  Critical reading and appraisal of the medical literature (Metz, Verbeek-Weel & Huisjes, 2001)  Apply certain theorems and laws of logic to clinical and research data in order to assess their validity and usefulness (Metz, Verbeek-Weel & Huisjes, 2001)  **Out of all other domains:**  Interprets medical evidence in a critical and scientific manner. (Carmichael & Hourn, 2011)  - Maintain critical thinking skills and use decision-support tools appropriately; (National Alliance for Physician Competence, 2009)  - engage in a review of the medical literature and other sources of medical information, evaluate the quality of evidence, assess its relevance to our specific needs, and integrate the information into our daily practice; (National Alliance for Physician Competence, 2009)  Accessing and evaluating information  We demonstrate scientific rigor in dealing with clinical situations. (National Alliance for Physician Competence, 2009)  - apply knowledge of study design and statistical methods to the appraisal of clinical studies and other information on diagnostic and therapeutic effectiveness; (National Alliance for Physician Competence, 2009)  Applying knowledge of scientific methodologies to critically evaluate research findings. (Scottish Deans' Medical Education Group, 2008  Recognizing the limitations of evidence-based medicine. (Scottish Deans' Medical Education Group, 2008  - Clinical reasoning  How to recognize and define the problem, analyze and interpret information, cope with limitations of information and personal limitations and find appropriate solutions. (Scottish Deans' Medical Education Group, 2008  - critically appraise published medical literature (Cumming & Ross, 2008)  The doctor is able to apply certain theorems and laws of logic to clinical and research data in order to assess their validity and usefulness (Metz, Verbeek-Weel & Huisjes, 2001)  - Critically appraises evidence and information (ACF Revision Working Group, 2009)  Evaluates the quality and relevance of information found using information technology. (Carmichael & Hourn, 2011)  critical appraisal of study methodology, internal and external validity of results: systematic error (selection bias, information bias); confounding and how to deal with it (age standardization, stratification, multivariable analysis). (Working Group of the Swiss Medical Schools, 2008)  - The ability to [...] manage and utilize biomedical information for solving problems and making decisions that are relevant to the care of individuals and populations (MSOP Report Writing Group, 1998)  How to […] analyze and interpret evidence. (Scottish Deans' Medical Education Group, 2008  Acquire, assess, apply and integrate new knowledge, | |
|  | | | | | |
| **Clinical Application** (n = 13)  quotes: 72 | **Research** (n = 11)  quotes: 50 | | **Lifelong Learning** (n = 13)  quotes: 54 | | **Teaching and Education** (n = 10)  quotes: 33 |
| **Knowledge about Clinical Application** (n = 7)  **Out of frameworks with a scholar-role:**  Describe the aetiology, pathology, clinical features, natural history and prognosis of common and important presentations at all stages of life. (AMC, 2013)  Explain the scientific bases for common disease presentation. (General Medial Council, 2009)  The physician demonstrates up to date knowledge of the evidence-based standard of medical care for common ailments and illnesses and of the basic urgent interventions. (Working Group of the Swiss Medical Schools, 2008)  **Out of all other domains:**  Awareness of how medical conditions/diseases are classified e.g. International Classification of Diseases (ICD). (Scottish Deans' Medical Education Group, 2008  An understanding of the power of the scientific method in establishing the causation of disease and efficacy of traditional and non-traditional therapies (Frank, Jabbour & CanMEDS Working Group, 2005)  Access and utilize components of the health care system and health delivery in a manner that is appropriate, cost effective, fair and in compliance with the national health care priorities and policies… (Medical Council of India, 2011)  We demonstrate up to date knowledge and skills about basic medical, clinical, and related sciences… (National Alliance for Physician Competence, 2009)  Demonstrate knowledge of national and regional health care policies including the national rural health mission, frameworks, economics and systems that influence health. Promotion, health care delivery, disease prevention, effectiveness, responsiveness, quality and patient safety. (Medical Council of India, 2011) | **Knowledge about Research**  (n = 7)  **Out of frameworks with a scholar-role:**  The physician demonstrates knowledge of general principles and methods of scientific medical research and their evolution over time. (Working Group of the Swiss Medical Schools, 2008)  The physician demonstrates knowledge of principles of […] basic statistics. (Working Group of the Swiss Medical Schools, 2008)  The physician demonstrates awareness of the scientific base and conceptual framework of modern medicine, its perception in society and its historical evolution over time. (Working Group of the Swiss Medical Schools, 2008)  Describe the principles of research and scholarly inquiry (Frank, Jabbour & CanMEDS Working Group, 2005)  He is acquainted with the basic principles of scientific research, not only in the form of scientific knowledge, but also in the form of practical experience through active participation in a scientific research project. (Metz, Verbeek-Weel & Huisjes, 2001)  The doctor has knowledge of:   - The general principles of hypothesis formation, methods ( including data gathering), concepts of scientific research relevant to medicine - The fundamentals of scientific methodology, biostatistics and epidemiology - The way of thinking in scientific research (through active participation) (Metz, Verbeek-Weel & Huisjes, 2001)   Apply theoretical frameworks (psychology, social sciences,...) (General Medial Council, 2009)  Demonstrate and understanding of established and evolving biological, clinical, epidemiological, social and behavioral sciences. (AMC, 2013)  Describe the principles of research ethics (Frank, Jabbour & CanMEDS Working Group, 2005)  Understand the ethical and governance issues involved in medical research. (General Medial Council, 2009)  **Out of all other domains:**  The physician identifies the ethical principles and values emerging in a given clinical situation or in a research project and identifies the conflicts of ethical principles and values. (Working Group of the Swiss Medical Schools, 2008)  We seek opportunities to add to the body of knowledge of medicine. When engaged in research, we:  - comply with established standards and appropriately credit ideas to their sources; (National Alliance for Physician Competence, 2009)  Understanding statistical principles. (Scottish Deans' Medical Education Group, 2008  Knowledge and appreciation of quantitative and qualitative methodologies, including the difference between them, and their appropriate usage. (Scottish Deans' Medical Education Group, 2008  Appreciation of the value of medical research and how this is organizes and funded. Knowledge of the potential role of research in career progression and the opportunities for research. (Scottish Deans' Medical Education Group, 2008  Awareness of progress in medical sciences and how it is achieved in the potential for every doctor to contribute to such progress. (Scottish Deans' Medical Education Group, 2008  - legal aspects of scientific research on humans (Metz, Verbeek-Weel & Huisjes, 2001)  Ethics: -the influence of scientific developments on standards and values  - ethics and medical scientific research (Metz, Verbeek-Weel & Huisjes, 2001) | | **Knowledge about and Attitude of Lifelong Learning** (n = 9)  **Out of frameworks with a scholar-role:**  - capacity to learn (including self-directed learning) (Cumming & Ross, 2008)  As Scholars, physicians demonstrate a lifelong commitment to reflective learning, as well as the creation, dissemination, application and translation of medical knowledge. Description: engage in a lifelong pursuit of mastering their domain of expertise (Frank, Jabbour & CanMEDS Working Group, 2005)  Describe the principles and strategies for implementing a personal knowledge management system (Frank, Jabbour & CanMEDS Working Group, 2005)  Describe the principles of maintenance of competence  As learners, they recognize the need to be continually learning and model this for others (Frank, Jabbour & CanMEDS Working Group, 2005)  They recognize the need to be continually learning. (Working Group of the Swiss Medical Schools, 2008)  At the end of undergraduate education and the beginning of postgraduate training physicians engage in a lifelong pursuit of mastery of their domain of professional expertise. (Working Group of the Swiss Medical Schools, 2008)  Lifelong learner committed to continuous improvement of skills and knowledge. (Medical Council of India, 2011)  responsibility for enhancing one’s own professional competence.  **The doctor demonstrates…**  - awareness of the need for continuing medical education and lifelong learning (Metz, Verbeek-Weel & Huisjes, 2001)  **Out of all other domains:**  Knowledge and application of the basic theories of learning and teaching. (Scottish Deans' Medical Education Group, 2008  Self-awareness  Conducting oneself as a reflective and accountable practitioner.  Making best use of sources of informed criticism such as assessment, audit and appraisal. Responding positively to assessment, audit, and appraisal. (Scottish Deans' Medical Education Group, 2008  Recognizing the importance of adopting a culture of lifelong learning and fostering this in the health service. (Scottish Deans' Medical Education Group, 2008  - An understanding of the needs to engage in lifelong learning to stay abreast of relevant scientific advances, especially in the disciplines of genetics and molecular biology (MSOP Report Writing Group, 1998)  - critical and self-critical abilities, reflective practice (Cumming & Ross, 2008)  He knows that to be able to continue to function adequately as a doctor, he will need to participate in continuing medical education and assessment […](Metz, Verbeek-Weel & Huisjes, 2001)  feel responsible for his own continuing education and take initiative in this respect (Metz, Verbeek-Weel & Huisjes, 2001)  …demonstrate lifelong learning behaviors ... (AMC, 2013)  Good physicians exhibit commitment to the ethical and professional standards of the medical profession. We:  **-** are committed to excellence and ongoing professional development; (National Alliance for Physician Competence, 2009) | | **Knowledge about Teaching and Education** (n = 4)  **Out of frameworks with a scholar-role:**  Describe principles of learning relevant to medical education (Frank, Jabbour & CanMEDS Working Group, 2005)  Describe the principles of ethics with respect of teaching (Frank, Jabbour & CanMEDS Working Group, 2005)  **Out of other domains:**  You should be willing to take on a mentoring role for more junior doctors and other healthcare professionals.  You must work in partnership with patients, sharing with them the information they will need to make decisions about their care including: any other information patients need if they are asked to agree to be involved in teaching or research.  Good physicians practice effectively in system of healthcare. We:  - recognize the value of teaching and training others. (National Alliance for Physician Competence, 2009)  Understanding the potential benefits to be gained by learning with and being taught by other healthcare professionals. (Scottish Deans' Medical Education Group, 2008  Recognizing the important role of all doctors as mentors and teachers. (Scottish Deans' Medical Education Group, 2008 |
| **Application of evidence to Patient Care** (n = 13)  **Out of frameworks with a scholar-role:**  - capacity for applying knowledge in practice (Cumming & Ross, 2008)  Through their scholarly activities, they contribute to the […] application and translation of medical knowledge (Frank, Jabbour & CanMEDS Working Group, 2005)  Apply social science principles, method and knowledge to medical practice. (General Medial Council, 2009)  Apply to medical practice the principles, methods and knowledge of population health and the improvement of health and health care. (General Medial Council, 2009)  Apply psychological principles, method and knowledge to medical practice. (General Medial Council, 2009)  The graduate will be able to apply to medical practice biomedical scientific principles, method and knowledge relating to: anatomy, biochemistry, cell biology, genetics, immunology, microbiology, molecular biology, nutrition, pathology, pharmacology and physiology. (General Medial Council, 2009)  Be able to apply newly gained knowledge or skills to the care of the patient. (Medical Council of India, 2011)  Apply core medical and scientific knowledge to individual patients, populations and health systems. (AMC, 2013)  The physician participates in the dissemination of knew knowledge and practices among colleagues and other collaborators and its translation into daily practice. (Working Group of the Swiss Medical Schools, 2008)  The physician shows ability to work with local / national guidelines and protocols and demonstrates a critical approach in doing this. (Working Group of the Swiss Medical Schools, 2008)  Integrate critical appraisal conclusions into clinical care (Frank, Jabbour & CanMEDS Working Group, 2005)  Integrate new learning into practice (Frank, Jabbour & CanMEDS Working Group, 2005)  Justify the selection of appropriate investigations for common clinical cases. (General Medial Council, 2009)  Apply findings from the literature to answer questions raised by specific clinical problems. (General Medial Council, 2009)  Evaluate and apply epidemiological data in managing health care for the individual and the community. (General Medial Council, 2009)  - Apply newly acquired insights; keep abreast of the medical literature (Metz, Verbeek-Weel & Huisjes, 2001)  The doctor is able to…  - approach a health problem in systematic way using:  - theories on hypothesis formation and problem-solving  - models  - decision theories (Metz, Verbeek-Weel & Huisjes, 2001)  **Out of all other domains:**  applications of epidemiology: descriptive and analytical approaches. (Working Group of the Swiss Medical Schools, 2008)  Formulate a plan for treatment, management and discharge, according to established principles and best evidence, in partnership with the patient, their careers, and other health professionals as appropriate. (General Medial Council, 2009)  …ensure that patients receive the highest level of professional care. (General Medial Council, 2009)  Applies the concept of specificity, sensitivity, pre and post test probability to the interpretation of common diagnostic procedures. (Carmichael & Hourn, 2011)  Applies information gained to optimize patient care. (Carmichael & Hourn, 2011)  Applies epidemiological data in managing the health of patients and their communities. (Carmichael & Hourn, 2011)  Be able to prescribe and safely administer appropriate therapies including nutritional interventions based on the principles of rational drug therapy, scientific validity, evidence and cost effectiveness that conform to establish national and regional health programs and policies for the following:   - Disease prevention - Health promotion and cure - Pain and distress alleviation - Rehabilitation and palliation (Medical Council of India, 2011)   Be able to choose the appropriate diagnostic test and interpret these tests based on scientific validity, cost effectiveness and clinical context. (Medical Council of India, 2011)  In providing clinical care you must:  b) provide effective treatments based on the best available evidence  You must be familiar with guidelines and developments that affect your work. Apply knowledge and experience to practice.  Apply the principles, skills and knowledge of evidence-based medicine´  - apply evidence to practice  - apply the principles, skills and knowledge of evidence-based medicine (Cumming & Ross, 2008)  - apply scientific principles, method and knowledge to medical practice… (Cumming & Ross, 2008)  - Uses best available evidence in clinical decision-making (ACF Revision Working Group, 2009)  - Applies the principles of evidence-based practice and hierarchy of evidence (ACF Revision Working Group, 2009)  - Establishes and uses current evidence based resources to support patient care and own learning (ACF Revision Working Group, 2009)  Using information in evidence-based practice. (Scottish Deans' Medical Education Group, 2008  Working with guidelines and protocols. (Scottish Deans' Medical Education Group, 2008  - Demonstrate the ability to implement, where appropriate, risk reduction strategies for individual patients (Scottish Deans' Medical Education Group, 2008  The use of evidence-based medicine and effective interventions. (Scottish Deans' Medical Education Group, 2008  Identifying and using professional guidelines. (Scottish Deans' Medical Education Group, 2008  … They also must understand the scientific basis and evidence of effectiveness for each of the therapeutic options that are available for patients at different times in the course of the patient’s conditions, and be prepared to discuss those options with patients in an honest and objective fashion. (MSOP Report Writing Group, 1998)  Physicians must understand the scientific basis of medicine and be able to apply that understanding to the practice of medicine. (MSOP Report Writing Group, 1998)  - [We] understand and are able to explain the limitations of medical knowledge, using our clinical judgment to provide care for patients when knowledge is insufficient. (National Alliance for Physician Competence, 2009)  We make informed decisions about diagnostic and therapeutic interventions based on patient information and preferences, up to date scientific evidence and clinical judgment. (National Alliance for Physician Competence, 2009)  - [We] provide or arrange for advice, investigations, or treatment based on available evidence and in accordance with our patient’s preferences and living circumstances, including those related to cost and cultural expectations, and our clinical judgment about likely effectiveness; (National Alliance for Physician Competence, 2009)  - [We] identify the patient’s most significant problems and diagnoses based on all available evidence and reach agreement with the patient on the priority of identified problems. (National Alliance for Physician Competence, 2009)  We adhere to establish guidelines and best practices.  - adhere to these guidelines or document a rational for deviating from them; (National Alliance for Physician Competence, 2009)  **Good physicians maintain knowledge and skills. We:**  - demonstrate up to date knowledge and the application of that knowledge to patient care and public health;  - seek and apply guidelines and best practices in making individual patient care decisions; (National Alliance for Physician Competence, 2009)  - [We] consider the information that patients bring about their conditions using evidence-based standards. (National Alliance for Physician Competence, 2009)  -work accordingly to guidelines whenever possible (Metz, Verbeek-Weel & Huisjes, 2001)  We [apply up to date knowledge and skills about basic medical, clinical, and related sciences to patient care and public health. (National Alliance for Physician Competence, 2009) | **Performing Research**  (n = 11)  **Out of frameworks with a scholar-role:**  Through their scholarly activities, they contribute to the creation, dissemination [… ]of medical knowledge (Frank, Jabbour & CanMEDS Working Group, 2005)  Contribute to the creation, dissemination, application, and translation of new medical knowledge and practices. (Frank, Jabbour & CanMEDS Working Group, 2005)  - research skills. (Cumming & Ross, 2008)  …contributes to the development, dissemination, and translation of new knowledge and practices (Working Group of the Swiss Medical Schools, 2008)  **Out of all other domains:**  - apply scientific principles, method and knowledge to […] research (Cumming & Ross, 2008)   - **Research question**   **Out of frameworks with a scholar-role:**  Formulate simple relevant research questions in biomedical science, psychosocial science or population science, and design appropriate studies or experiments to address the questions. (General Medial Council, 2009)  Be able to develop a research question and be familiar with basic, clinical and translational research as it applies the care of the patient. (Medical Council of India, 2011)  Pose a scholarly question (Frank, Jabbour & CanMEDS Working Group, 2005)  Apply knowledge of common scientific methods to formulate relevant research questions… (AMC, 2013)   - **Methods/Study design**   **Out of frameworks with a scholar-role:**  […] applicable study designs. (AMC, 2013)  Apply scientific method and approaches to medical research.  Select and apply appropriate methods to address the question (Frank, Jabbour & CanMEDS Working Group, 2005)  **Out of all other domains:**  …type of variables: categorical (binary, nominal, ordinal) and numerical (discrete, continuous). (Working Group of the Swiss Medical Schools, 2008)  …observational and experimental study designs, their advantages and disadvantages and areas of application: case series cross-sectional studies, case-control studies, cohort studies, randomized controlled trials, systematic reviews, meta-analysis. (Working Group of the Swiss Medical Schools, 2008)  Innovative use of knowledge, techniques, technologies and methodologies. (Scottish Deans' Medical Education Group, 2008  Choosing the appropriate statistical test for a given situation. (Scottish Deans' Medical Education Group, 2008   - **Analysis**   **Out of frameworks with a scholar-role:**  - capacity for analysis and synthesis (Cumming & Ross, 2008)  **Out of all other domains:**  describing data and their variability: frequency, proportion, mean, standard deviation, median, histogram, box-plot, scatter plot, survival curve. (Working Group of the Swiss Medical Schools, 2008)  estimation: standard errors (of a mean, difference between two means proportion, difference between two proportions), confidence intervals. (Working Group of the Swiss Medical Schools, 2008)  hypothesis testing: null and alternative hypotheses, interpretation of P values, relation between P values and confidence intervals. (Working Group of the Swiss Medical Schools, 2008)  measures of association: relative measures (risk ratio, rate ratio, odds ratio); absolute measures (risk difference, number needed to treat / harm, attributable risk). (Working Group of the Swiss Medical Schools, 2008)  Interpreting the outcomes of statistical analyses. (Scottish Deans' Medical Education Group, 2008   - **Dissemination**   **Out of frameworks with a scholar-role:**  Appropriately disseminate the findings of a study. (Frank, Jabbour & CanMEDS Working Group, 2005)  The doctor is able to…  - report, interpret and evaluate research (Metz, Verbeek-Weel & Huisjes, 2001)   - **Team**   **Out of frameworks with a scholar-role:**  The physician shows readiness and ability to participate in collaborative research projects. (Working Group of the Swiss Medical Schools, 2008)  Participates effectively in research activities. (Carmichael & Hourn, 2011)   - **Ethics**   **Out of all other domains:**  - [We] protect the interest of research subjects as a first priority if we are involved in research involving human subjects; (National Alliance for Physician Competence, 2009)  We are satisfied that we have consent or other authority before we undertake any examination or investigation, provide treatment or involve patients in teaching or research; (National Alliance for Physician Competence, 2009)  You must act with honesty and integrity when designing, organizing or carrying out research, and follow national research governance guidelines and our guidance.  You must be honest and trustworthy when writing reports, and when completing or signing forms, reports and other documents. You must make sure that any documents you write or sign are not false or misleading.  a) You must take reasonable steps to check the information is correct.  b) You must not deliberatively leave out relevant information.  You must be satisfied that you have consent or other valid authority before you carry out any examination or investigation, provide treatment or involve patients or volunteers in teaching or research.  Record your work clearly, accurately and legibly.  Knowledge and understanding of the legal and ethical framework within which medical research is conducted. This should include:   - The distinction between audit and research - The requirement for ethical approval of research, and where to find information and documentation if needed - The need to protect the rights of patients and volunteers participating in research, in relation to recruitment, consent, confidentially, patient safety, data handling and compensation arrangements - The potential consequences of research malpractice   - Patient consent:  Consent for research. (Scottish Deans' Medical Education Group, 2008 | | **Learning causes** **incl. Self-Assessment** (n = 9)  **Out of frameworks with a scholar-role:**  Pose an appropriate learning question (Frank, Jabbour & CanMEDS Working Group, 2005)  Recognize and reflect learning issues in practice (Frank, Jabbour & CanMEDS Working Group, 2005)  The physician regularly assesses personal learning, recognizes errors and attempts too learn from them. (Working Group of the Swiss Medical Schools, 2008)  The physician strives to evaluate the outcome of learning her/his practice. (Working Group of the Swiss Medical Schools, 2008)  Be able to perform an objective self-assessment of knowledge and skills and continue learning… (Medical Council of India, 2011)  Be able to introspect and utilize experiences to enhance personal and professional growth and learning. (Medical Council of India, 2011)  - identify blind spots and/or gaps in his professional practice… (Metz, Verbeek-Weel & Huisjes, 2001)  **Out of all other domains:**  …learn to adapt to changing circumstances…. (General Medial Council, 2009)  Reflect, learn and [….](General Medial Council, 2009)  We ensure that our scope of practice remains within our own competence.  - We: are aware of the boundaries of our knowledge and skills; (National Alliance for Physician Competence, 2009)  We:   - use information about our own patients and larger populations from which our patients are drawn to guide our learning; - engage in periodic reassessment to evaluate improvement and to direct continued learning; - engage in a systematic program of self-assessment of our medical knowledge and skills; - participate in ongoing, practice-specific assessment of our own knowledge and skills; (National Alliance for Physician Competence, 2009)   - Identifies and addresses personal learning objectives (ACF Revision Working Group, 2009)  - Seeks opportunities to reflect on and learn from clinical practice (ACF Revision Working Group, 2009)  - The capacity to recognize and accept limitations in one’s knowledge and clinical skills and a commitment to continuously improve one’s knowledge and ability. (MSOP Report Writing Group, 1998)  Managing one’s own learning and keep up to date as demonstrated by:   - Recognizing limitations of current personal understanding and capabilities and identifying areas and strategies for development (Scottish Deans' Medical Education Group, 2008 | | **Teaching Skills:** (n = 9)   - **Teaching**   **Out of frameworks with a scholar-role:**  Facilitate the learning of patients, families, students, residents, other health professionals, the public and others, as appropriate (Frank, Jabbour & CanMEDS Working Group, 2005)  Collaboratively identify the learning needs and desired learning outcomes of others (Frank, Jabbour & CanMEDS Working Group, 2005)    Select effective teaching strategies and content to facilitate others’ learning (Frank, Jabbour & CanMEDS Working Group, 2005)  Demonstrate an effective lecture or presentation (Frank, Jabbour & CanMEDS Working Group, 2005)  As teacher, they facilitate the education of their students, patients, colleagues and others. (Frank, Jabbour & CanMEDS Working Group, 2005)  […] communicate information to others. (Metz, Verbeek-Weel & Huisjes, 2001)  …facilitate the learning of patients, students and other health professionals. (Working Group of the Swiss Medical Schools, 2008)  The physician shows basic teaching skills in presentation for students and other health professionals. (Working Group of the Swiss Medical Schools, 2008)  **Out of all other domains:**  You should be prepared to contribute to teaching and training doctors and students.  [..] teach others.  […] skills in educating colleagues. (AMC, 2013)  Contributes to the professional education of peers and junior colleagues. (Carmichael & Hourn, 2011)  An appreciation of the responsibility to contribute towards the generation of knowledge and the professional education of junior colleagues. (Carmichael & Hourn, 2011)  We facilitate the learning of students and graduate physicians and / or other healthcare professionals when in a position to do so. If we are involved in teaching, we develop the skills, attitudes and practices necessary to provide competent training and evaluation for current and future healthcare professionals. We ensure that all staff members, students, and residents for whom we are responsible are properly supervised. (National Alliance for Physician Competence, 2009)  giving teaching presentations to students and other health professionals (Working Group of the Swiss Medical Schools, 2008)  - Plans, develops and conducts teaching sessions for peers and juniors. (ACF Revision Working Group, 2009)  - Uses varied approaches to teaching small and large groups (ACF Revision Working Group, 2009)  - Incorporates teaching into clinical work (ACF Revision Working Group, 2009)   - **Feedback**   **Out of frameworks with a scholar-role:**  Provide effective feedback. (Frank, Jabbour & CanMEDS Working Group, 2005)  …provides constructive feedback (Working Group of the Swiss Medical Schools, 2008)  **Out of all other domains:**  - Seeks and responds to feedback on learning (ACF Revision Working Group, 2009)  - Evaluates and responds to feedback on own teaching (ACF Revision Working Group, 2009)  6. Seeks feedback from peers, tutors, patients and colleagues. (Carmichael & Hourn, 2011)  Having the ability to appraise the performance of others. (Scottish Deans' Medical Education Group, 2008   - **Evaluation**   **Out of frameworks with a scholar-role:**  Assess and reflect on a teaching encounter (Frank, Jabbour & CanMEDS Working Group, 2005)  The physician assesses and reflects on a teaching encounter… (Working Group of the Swiss Medical Schools, 2008) |
| **Evaluation** / **Quality Management** (n = 8)  **Out of frameworks with a scholar-role:**  Describe measurement methods relevant to the improvement of clinical effectiveness and care. (General Medial Council, 2009)  Conduct a personal practice audit (Frank, Jabbour & CanMEDS Working Group, 2005)  Evaluate the impact of any change in practice (Frank, Jabbour & CanMEDS Working Group, 2005)  **Out of all other domains:**  - commitment to maintaining good practice, concern for quality (Cumming & Ross, 2008)  Leader and member of the health care team and system with capabilities to collect analyze and synthesize health data. (Medical Council of India, 2011)  …be able to collect, analyze and utilize health data (Medical Council of India, 2011)  We thoughtfully assess our own patient care practices, assimilate scientific evidence, and seek always to improve our patient care practices. (National Alliance for Physician Competence, 2009)  - We: apply the outcome of audits, appraisals and performance reviews to our practice;  - apply best practices and available benchmarks to our own patient care (National Alliance for Physician Competence, 2009)  Recognizing the link between evidence-based medicine and audit and the reasons for variation in clinical practice. (Scottish Deans' Medical Education Group, 2008  Understanding how routinely collected health information is used in service planning and delivery of care. (Scottish Deans' Medical Education Group, 2008  Self-evaluate their own professional practice (AMC, 2013)  - Participates in research and quality improvement activities where possible (ACF Revision Working Group, 2009)  We adhere to establish guidelines and best practices.  - We: regularly review establishes evidence-based practice guidelines germane to the scope of our practice; (National Alliance for Physician Competence, 2009)  - [We] promptly modify our practice to incorporate evidence-based improvements in care. (National Alliance for Physician Competence, 2009)  Contribute to the enhancement of quality care and patient safety in their practice, integrating the available best evidence and best practices (Frank, Jabbour & CanMEDS Working Group, 2005) |  | | **Learning** **Activities**  (n = 11)  **Out of frameworks with a scholar-role:**  Physicians are able to… Maintain and enhance professional activities through ongoing learning. (Frank, Jabbour & CanMEDS Working Group, 2005)  Be able to […] refine existing skills and acquire new skills. (Medical Council of India, 2011)  … try to eliminate [blind spots and/or gaps] (by means of continuing medical education courses and otherwise) (Metz, Verbeek-Weel & Huisjes, 2001)  Document the learning Process (Frank, Jabbour & CanMEDS Working Group, 2005)  **Out of all other domains:**  - assimilate evidence from scientific studies related to our patients’ health problems; (National Alliance for Physician Competence, 2009)  We apply the basic and clinically supportive sciences and skills that are appropriate to our scope of practice in the context of the best available medical evidence.  We:   - take personal responsibility of maintaining up to date knowledge of the basic science and clinical medicine and up-to-date clinical skills in areas relevant to our practice. - Complete appropriate training before undertaking new procedures or practices. - Participate regularly in learning activities that are relevant to our practice. - Take part regularly in learning activities that maintain and advance our competence and 432 (?) performance. (National Alliance for Physician Competence, 2009) - We develop individual learning plans that focus on areas of weakness; - in order to learn and improve, we take whatever advantage we can of information technology manage information about our patients; access medical information relevant to our practice; support our own education; (National Alliance for Physician Competence, 2009)   Good physicians actively learn from their practices. We:   - assimilate scientific evidence; - seek always to improve patient care practices. (National Alliance for Physician Competence, 2009)   You must regularly take part in activities that maintain and develop your competence and performance. (AMC, 2013)  Identifies learning activities that will enable him/her to address learning goals. (Carmichael & Hourn, 2011)  They must engage in lifelong learning to remain current in their understanding of the scientific basis of medicine. (MSOP Report Writing Group, 1998)  Undertakes appropriate learning activities. (Carmichael & Hourn, 2011)  Undertakes teaching, including development of teaching skills as part of professional development. (Carmichael & Hourn, 2011)  Demonstrates self directed learning. (Carmichael & Hourn, 2011)  Teaching, Learning and supervision:  Self-directed learning (ACF Revision Working Group, 2009)  Apply lifelong learning skills of the Scholar Role to implement a personal program to keep up-to-date, and enhance areas of professional competence. (Frank, Jabbour & CanMEDS Working Group, 2005)  Establish the foundations for lifelong learning and continuing professional development, including a professional development portfolio containing reflections, achievements and learning needs. (General Medial Council, 2009)  Is flexible: responds adequately to new developments.  The doctor can be expected to adapt his knowledge, skills and attitude to changes in health care, to scientific and social possibilities and developments, and to economic, legal and ethical limitations. He should keep abreast of these developments. (Metz, Verbeek-Weel & Huisjes, 2001)  You must keep your professional knowledge and skills up to date.  Managing one’s own learning and keep up to date as demonstrated by:   - Managing time, learning plans and activities for effective personal study, taking into account both academic achievement and individual professional development - Searching out, selecting and using appropriate learning resources, including technical aids - Employing appropriate and effective study techniques (Scottish Deans' Medical Education Group, 2008   Understanding the role and making appropriate use of log books and portfolios. (Scottish Deans' Medical Education Group, 2008  - Personal record keeping for professional development (Scottish Deans' Medical Education Group, 2008 | |  |

n = *number of outcome frameworks contributing per component or specification
code:*

- **Accreditation Standard for Primary Medical Education Providers and their Program of Study and Graduate Outcome Statement**

Australia / New Zealand

(AMC, 2013)

- **Developing a Framework of Competencies for Medical Graduate Outcomes**
  Australia/New Zealand

(Carmichael & Hourn, 2011)

- **The Scottish Doctor: Learning Outcomes for the Medical Undergraduate in Scottland: a Foundation for Competent and Reflective Praticioneers**Scotland
  (Scottish Deans' Medical Education Group, 2008)
- **CanMEDS Framework**Canada
  (Frank, Jabbour & CanMEDS Working Group, 2005)
- **Tomorrows Doctors. Outcomes and standards for undergraduate medical education**

United Kingdom
(General Medial Council, 2009)

- **Australian Curriculum Framework for** **Junior Doctors**

Australia / New Zealand
(ACF Revision Working Group, 2009)

- **Blueprint 2001: Training of doctors in the Netherlands**
  Netherlands
  (Metz, Verbeek-Weel & Huisjes, 2001)
- **The Tuning Project, learning Outcomes/Competencies for Undergraduate Medical Education in Europe**Europe
  (Cumming & Ross, 2008)
- **Report 1 – Learning Objectives for Medical Student Education, Guidelines for Medical Schools**USA
  (MSOP Report Writing Group, 1998)
- **Visions 2015**India
  (Medical Council of India, 2011)
- A **Guide to Good Medical Practice**
  USA
  (National Alliance for Physician Competence, 2009)
- **Good Medical Practice**UK
  (General Medical Council, 2013)
- **Swiss Catalogue of Learning Objectives for Undergraduate Medical Training**
  Switzerland
  (Working Group of the Swiss Medical Schools, 2008)
